# Supplementary material for: A molecular inventory of the faecal microbiomes of 23 marsupial species
Source: Microb Genom. 2026 Jan 9;12(1):001601. doi: 10.1099/mgen.0.001601 (PMC12788396; doi:10.1099/mgen.0.001601)
Supplement: Uncited Supplementary Material 1. [file mgen-12-01601-s001.pdf]

Supplementary Figures

A molecular inventory of the faecal microbiomes of 23 marsupial species

Kate L. Bowerman[1*^], Rochelle M. Soo[1*], Pierre-Alain Chaumeil[1], Michaela D. J. Blyton[1], Mette Sørensen[2], Disan Gunbilig[2], Maika Malig[4], Moutusee Islam[4], Julian Zaugg[1], David L. A. Wood[3], Ivan Liachko[4], Benjamin Auch[4], Mark Morrison[5], Lutz Krause[3], Birger Lindberg Møller[2], Elizabeth H. J. Neilson[2] and Philip Hugenholtz[1^]

1. Australian Centre for Ecogenomics, School of Chemistry & Molecular Biosciences, The University of Queensland, Brisbane, Australia
2. Department of Plant and Environmental Sciences, University of Copenhagen, Denmark
3. Microba Life Sciences, Brisbane, Queensland, Australia
4. Phase Genomics Inc., Seattle, WA, USA
5. Frazer Institute, School of Medicine, The University of Queensland, Brisbane, Queensland, Australia

*Equal contribution

^Corresponding: k.bowerman@uq.edu.au, p.hugenholtz@uq.edu.au

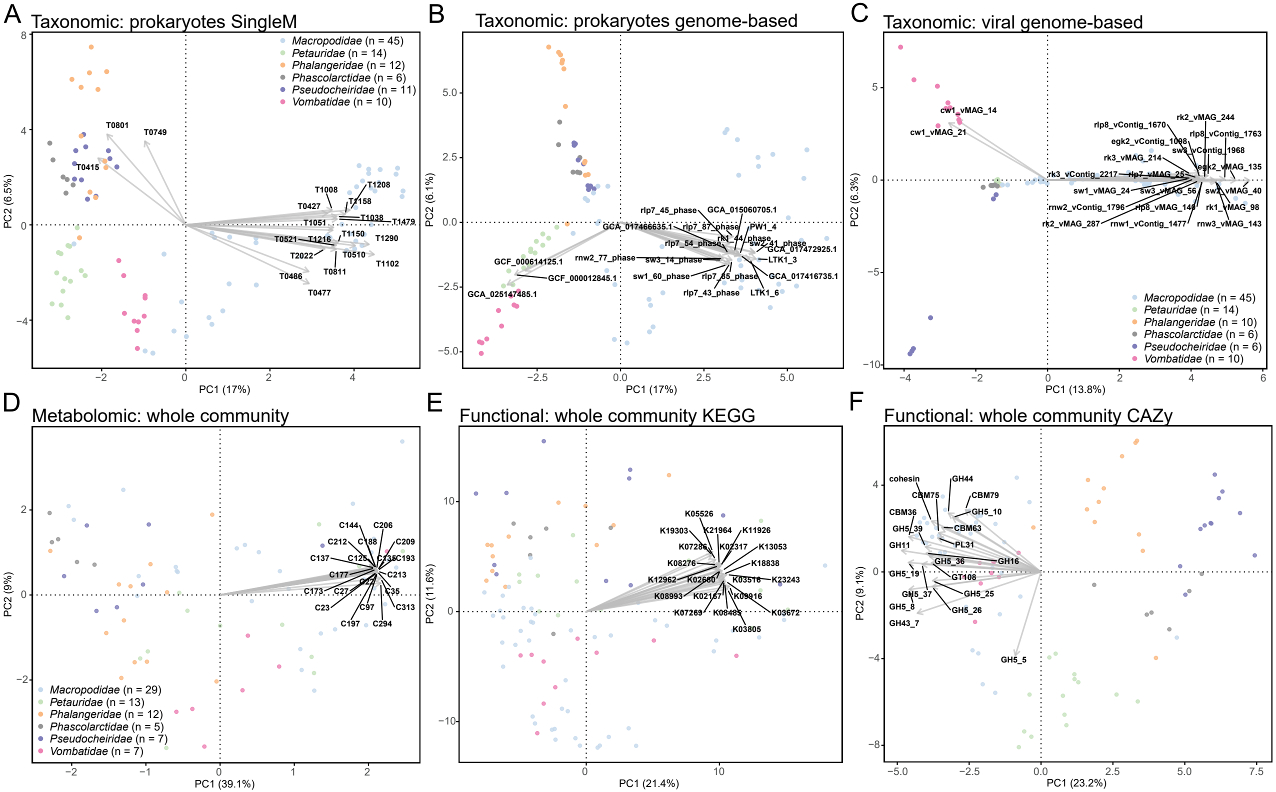

Fig. S1. Principal component analysis based on **a**, marker gene-based prokaryotic community **b**, genome-based prokaryotic community **c**, genome-based viral community **d**, metabolomic **e**, KEGG-based functional and **f**, CAZy-based functional profiles. Top 20 features based on Euclidean magnitude across PC1 and PC2 are displayed. Full lists are contained in Tables S4-S9. Analysis based on a single sample per animal (metagenome: n = 98; metabolome: n = 73). Samples with no viral mapping excluded (n = 7).

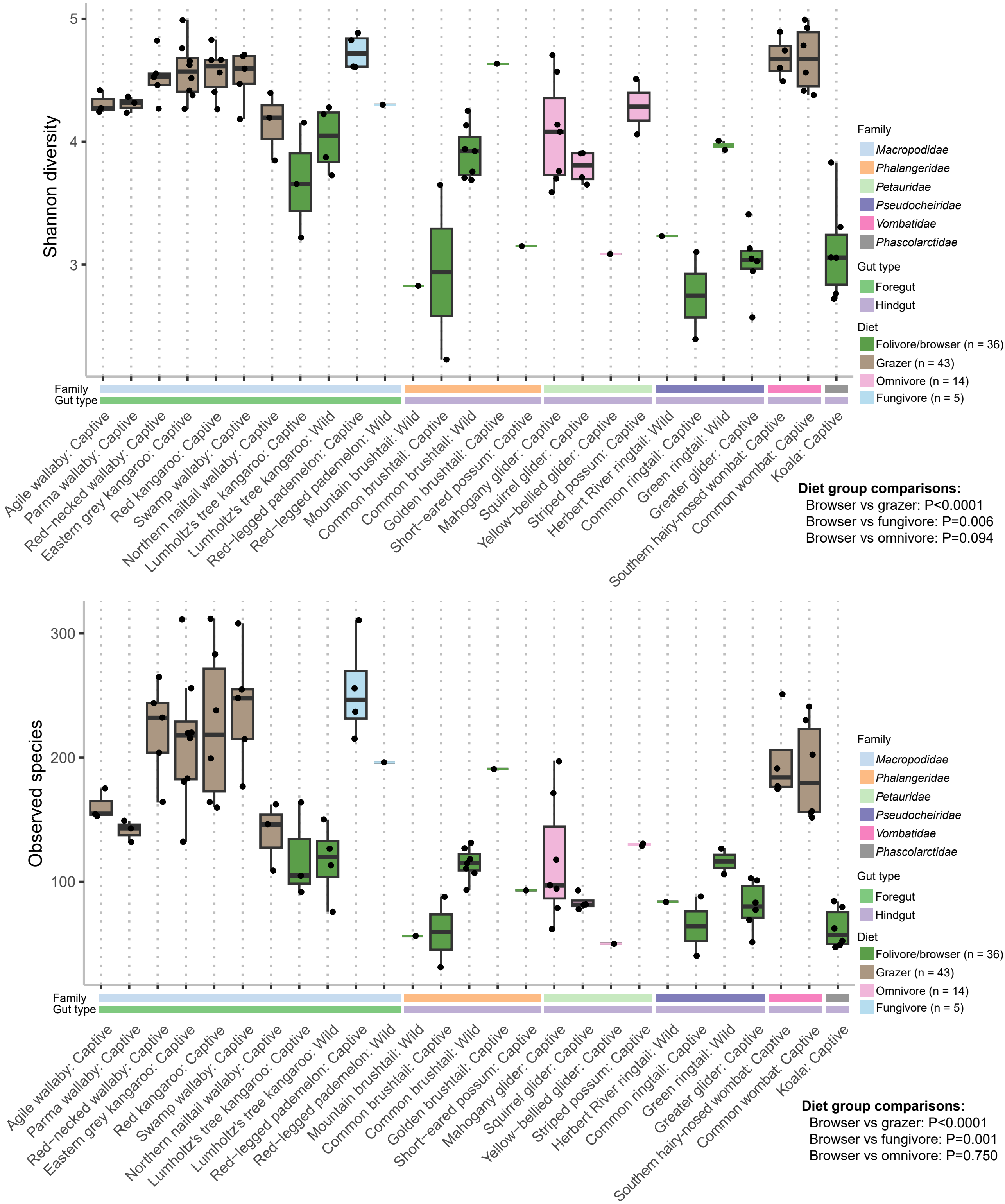

Fig. S2. Shannon diversity and observed species counts based on SingleM prokaryotic community profiles. Diet comparison based on classifications indicated in **Table S2** with significance determined using a linear mixed-effects model with squared Shannon and square-root transformed observed richness and species as a random effect. Analysis based on a single sample per animal (n = 98).

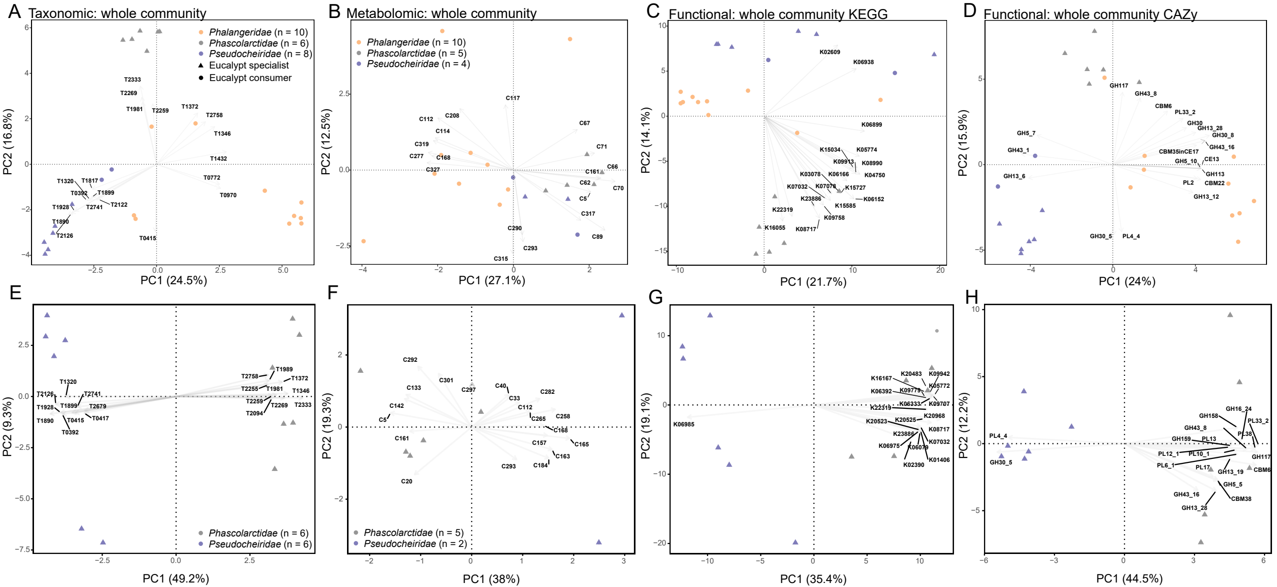

Fig. S3. Principal component analysis of eucalypt consuming marsupials (**a-d**) and eucalypt specialist marsupials (**e-h**) based on **a & e**, marker gene-based prokaryotic community, **b & f**, metabolomic, **c & g**, KEGG-based functional, **d & h**, CAZy-based functional profiles. Eucalypt consumers includes koalas, greater gliders, common and golden brushtails and common ringtails. Eucalypt specialists include koalas and greater gliders. Top 20 features based on Euclidean magnitude across PC1 and PC2 are displayed. Full lists are contained in Tables S10-S17. Analysis based on a single sample per animal.

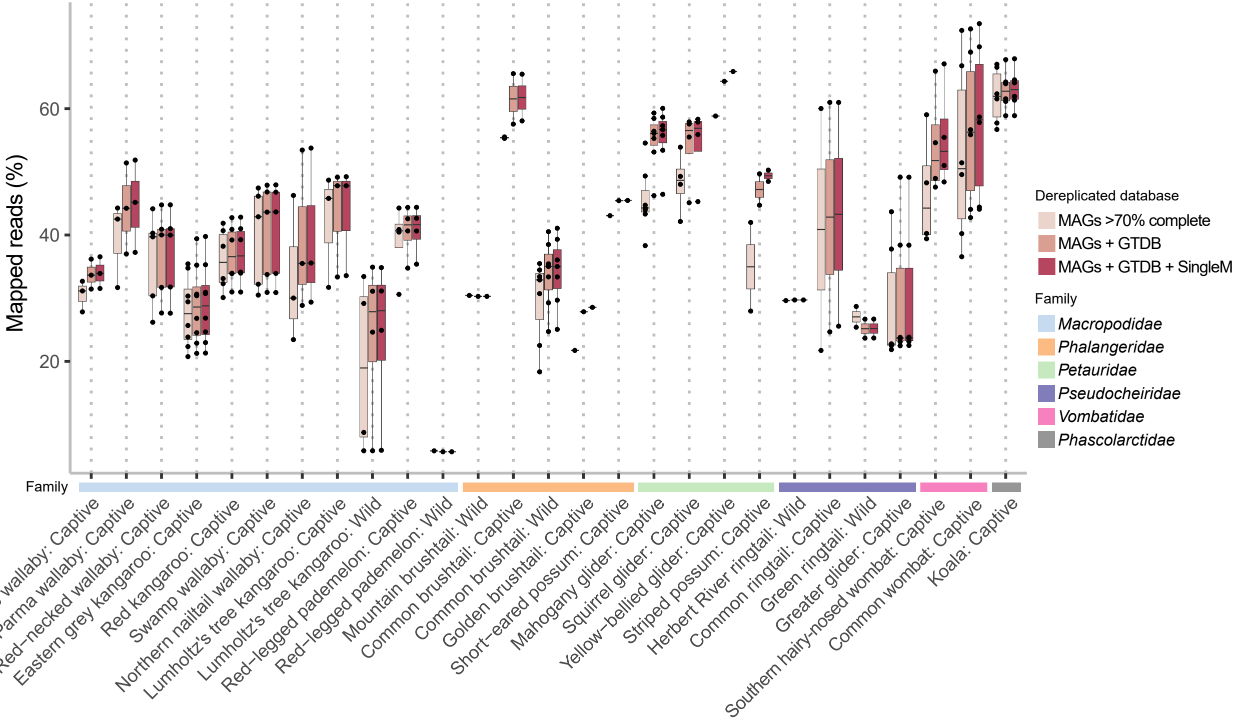

Fig. S4. Read recruitment to MAG database incorporating MAGs from the current study ≥70% complete with ≤5% contamination, or study MAGs combined with public genomes selected based on read mapping or marker gene-based profiles. Read mapping proportions based on filtered alignments with ≥95% identity across ≥90% of the read length.

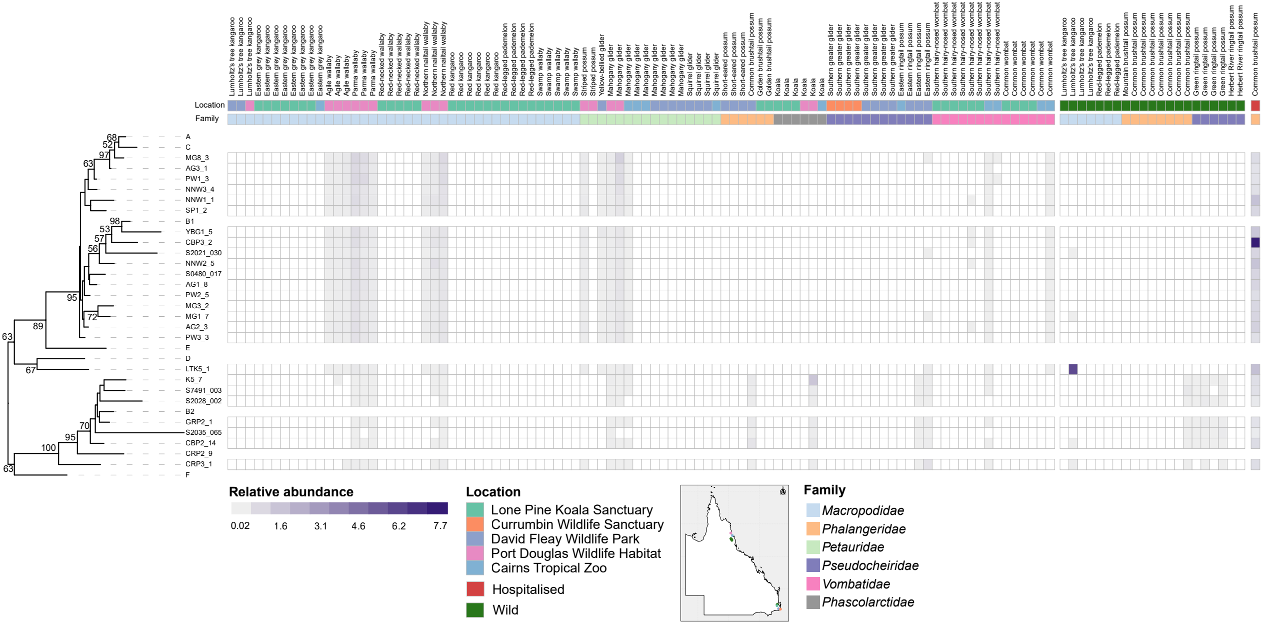

Fig. S5. Maximum-likelihood phylogenetic tree of *Escherichia coli* MAGs assembled from the dataset based on concatenated alignment of 15 marker genes (Wirth et al., 2006; Jaureguy et al., 2008). Reference genomes of each phylogroup are labelled A - F. Heatmap displays relative abundance per sample, based on read mapping. Bootstrap support derived from 1,000 replicates.

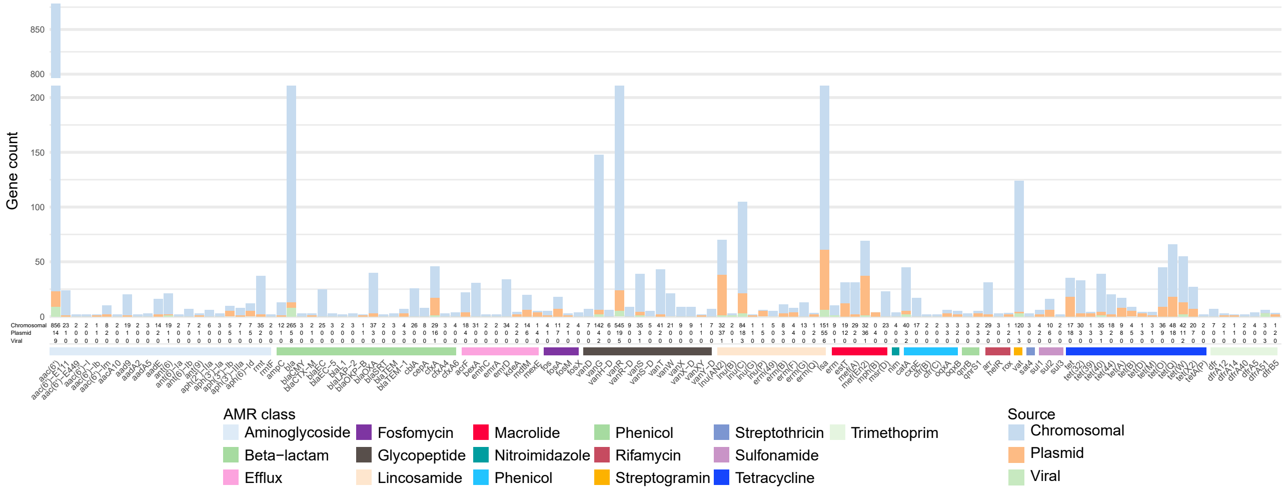

Fig. S6. Count of AMR genes identified on all contigs based on AMRFinder search, excluding singletons. Source indicates the geNomad-predicted sequence type of the contig carrying the representative gene sequence.

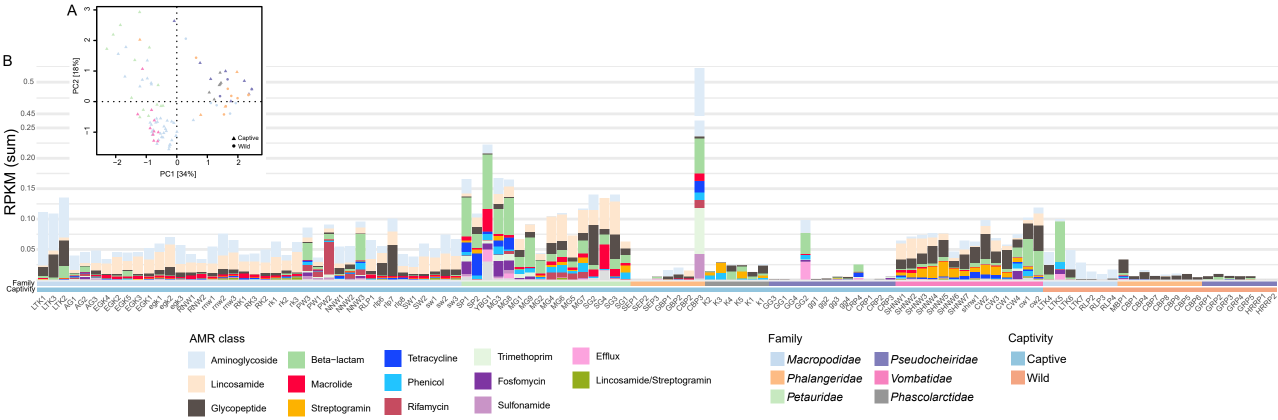

Fig. S7. a PCA based on AMR gene RPKM values. **b** RPKM sum per AMR class per marsupial faecal sample.

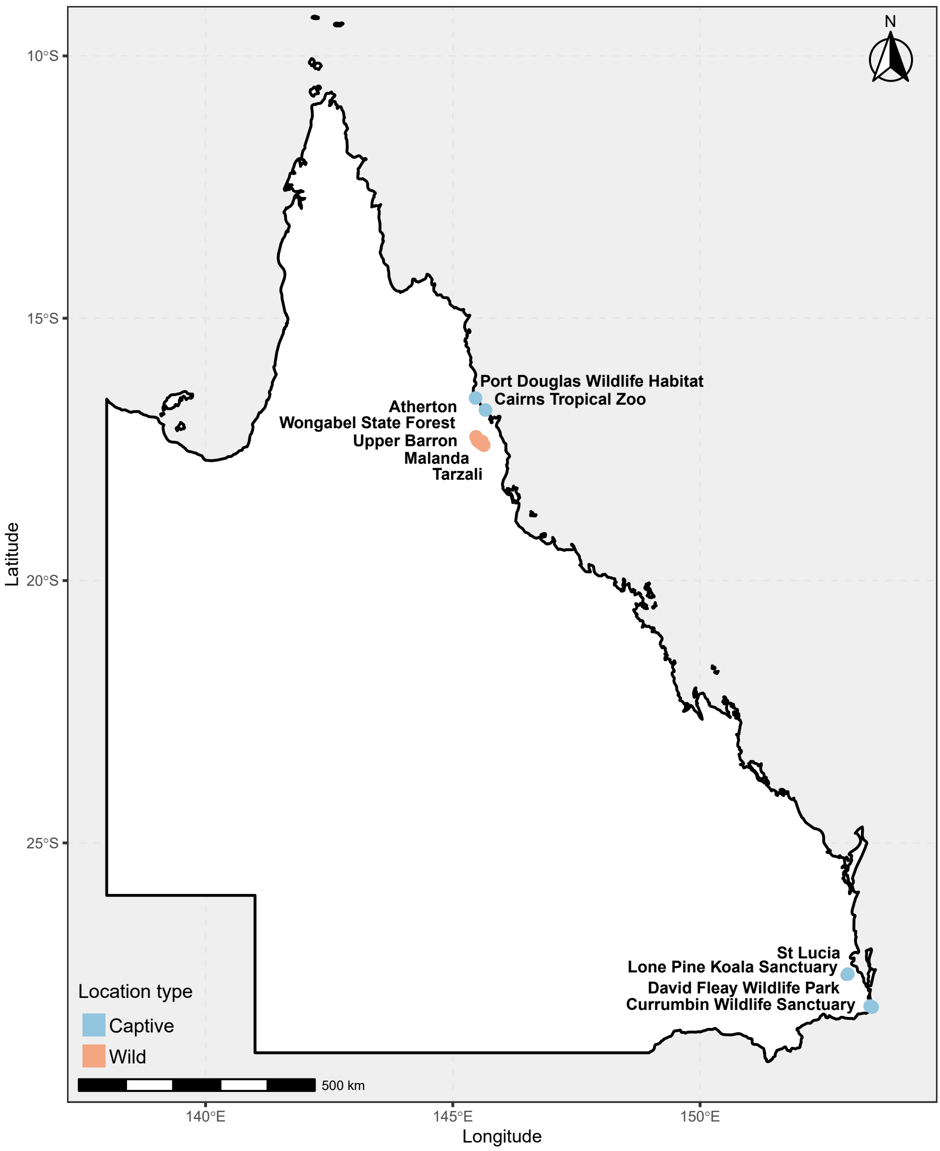

Fig. S8. Geographic locations of sample sites in Queensland, Australia.

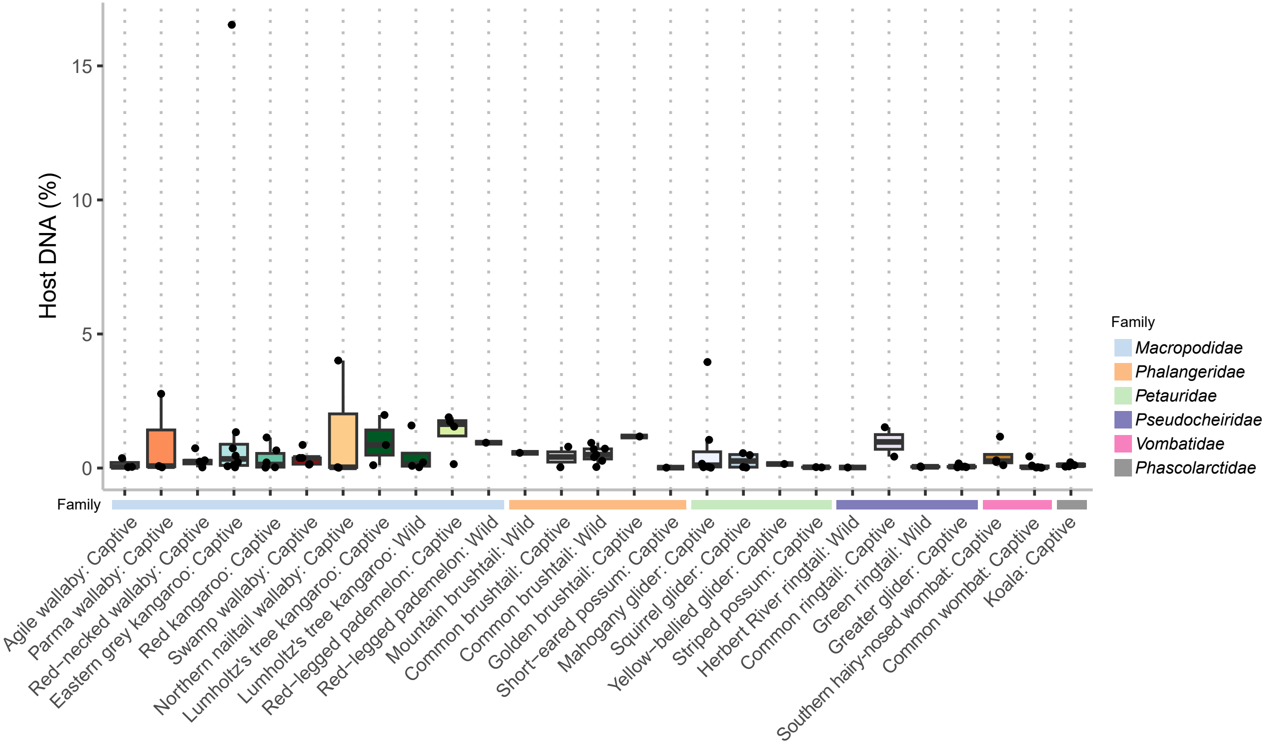

Fig. S9. The relative amount of host animal DNA in the faecal metagenomes was estimated by mapping sequencing reads to publicly available marsupial host genomes, including an exon capture dataset obtained from NCBI Genbank (Benson et al., 2013). As found in other animals, host contamination was low (<4% of total reads; Ong et al., 2022), with one conspicuous outlier from an eastern grey kangaroo (~17%) that may indicate elevated gut epithelial cells in the feces due to inflammation or other dysbiosis (Jiang et al., 2020).
